# Volar Dislocation of Second, Third and Fourth Carpometacarpal Joints in Association with a Bennet's Fracture of the Thumb *Carpo-Metacarpal Dislocation: A Case Report*

**DOI:** 10.2174/1874325001711011035

**Published:** 2017-08-29

**Authors:** Pierluigi Di Felice Ardente, Joan Camí Biayna, Jordi Saus Sarrias, Antoni Nuñez Muñoz, Guillem Figueras Coll, Patricio Vergara

**Affiliations:** Department of Orthopaedic Surgery, Hospital Sant Joan de Deu de Manresa (Fundació Althaia Xarxa Assistencial Universitària de Manresa), C/ Dr.Joan Soler, 1-3, 08243 Manresa, Barcelona, Spain

**Keywords:** Carpometacarpal joint, Dislocation, Volar, Emergency, Wound, Bennet’s fracture

## Abstract

Injuries of the carpometacarpal joints of the long fingers are infrequent and go often unnoticed if a thorough clinical exploration and complete radiological assessment is not carried out. The overall frequency of carpometacarpal joints injuries is of 1-2% among trauma of the wrist and the carpus. These are normally secondary to high energy trauma (such as a car or a motorbike accidents). Among these kinds of injuries, the volar dislocation of the metacarpals is much less frequent than the dorsal dislocation, being even rarer the cases in which the three middle metacarpals are involved.

In this case report, we present a case of volar dislocation of the middle three carpometacarpal joints in association with a Bennet’s fracture of the thumb in a 30-year-old male. He was treated in the operating room with reduction and stabilization using Kirschner wires, which allowed a satisfactory recovery of the mobility of the fingers eight weeks after the intervention. It is important to produce an admission diagnosis of this kind of injuries to be able to treat them immediately. Treatment must be performed quickly to reduce and stabilize the dislocation, since this will avoid the vasculo-nervous compression or the edema which increases cutaneous suffering and the risk of complications.

## 1 INTRODUCTION

Dislocation of the carpometacarpal joints is a very rare event and it is commonly linked with a high energy trauma [1]. The cases in which the three central metacarpals are affected and present a volar dislocation are even fewer [2]. Moreover, these might or might not be associated with Bennett's fracture [3]. These injuries are often difficult to diagnose in emergency departments if a proper radiological study is not carried out [4]. Dislocations of the carpometacarpal (CMC) joints often have subtle radiographic findings that may be overlooked in an acute setting and in the emergency room. Therefore, it is relevant to generate the appropriate views [5] to-allow for a quick diagnosis and treatment, thus reducing the risk of complications. Lateral and oblique views are important for the recognition of the true extent of the injury. Despite these facts, these kinds of injuries-commonly have good prognosis after the acute treatment, presenting a limited amount of complications. Early surgical intervention can lead to good functional results.

## 2 CASE REPORT

**Fig. (1):**
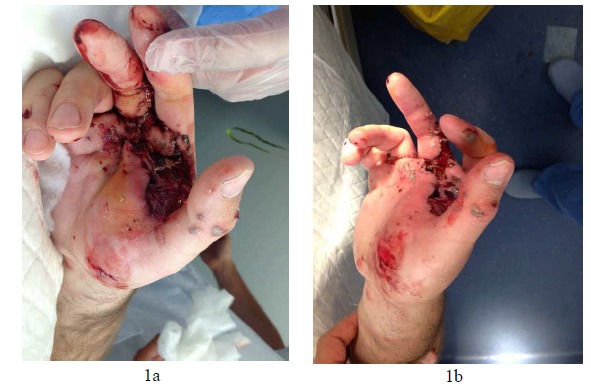
Edema and swelling of the hand with the presence of an important laceration at a thenar level.

**Fig. (2):**
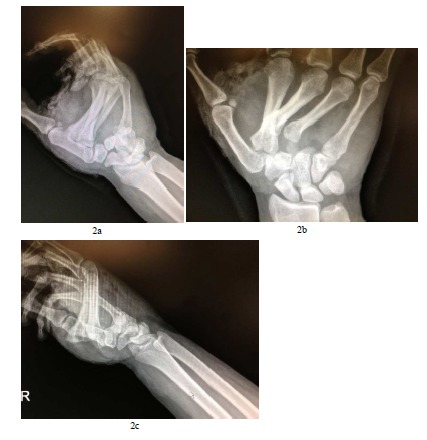
Postinjury radiographs (anteroposterior, lateral, and oblique views) showing volar and radial dislocation of the second, third, and fourth carpometacarpal joints.

Our case deals with a 33 years old male with no relevant medical background, who was admitted to the emergency department after a traffic accident. On his arrival, the patient was conscious and oriented, and presented a head trauma. He also presented a mechanism of direct contusion on his (non-dominant) right hand against the dashboard. In the physical examination, pain and a functional impotence of the hand at 45º was identified, with the presence of a blunt laceration at a thenar level (Figs. **1a**, **b**). Likewise, there was a large edema and swelling on the dorsal side of the hand. The distal vasculo-nervous structures remained intact. The diagnostic hypothesis was confirmed radiologically. This evidenced a radio volar dislocation on the second, third and fourth carpometacarpal joints, linked with a Bennett’s fracture of the first finger (Figs. **2a**, **b**, **c**). After the initial treatment and cleaning of the injuries at the emergency department, the patient was taken to the operating room immediately.

With the patient in supine position and under anesthesia, a thorough debridement of the wound was performed. The integrity of the superficial palmar arch and the rest of tendon structures present on that level were identified (Fig. **3a**). Secondly, with the elbow extended, a traction of the three middle fingers was performed, pressing the base of the metacarpals from volar to dorsal. Under scopic control, the correct reduction of the metacarpals was checked before proceeding with the osteosynthesis through multiple 1.5 mm Kirschner wires percutaneously. Taking into account the instability, the first wire was fixed in an oblique direction from the base of the first to the second metacarpal, stabilizing the radial side. Afterwards, the other wires were also fixed: one transversally and two longitudinally, following the axis of the central metacarpals Fig. **3b**.

**Fig. (4):**
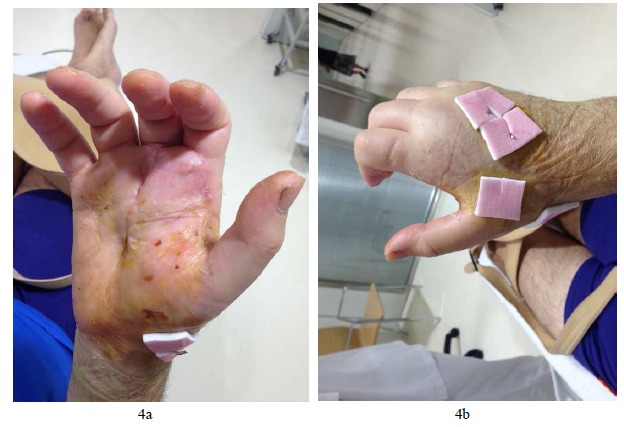
Evolution of the wounds on the fifth week aiming at correct healing and status of the wounds.

The post-operative radiographs showed the reduced second, third, and fourth carpometacarpal joints stabilized with Kirschner wires (Fig. **3c**). Daily treatment of the wounds and mobilization of the metacarpo-phalangeal joints was started the day after the operation to avoid a limitation of the mobility of the fingers secondary to prolonged immobilization. Six weeks later, the Kirschner wires were removed radiologically and the correct carpometacarpal stability was checked clinically. The wounds exhibited correct healing (Figs. **4a**, **b**) and eight weeks later the patient was able to move his fingers without pain and with a strength comparable to that of the other hand.

## 3 DISCUSSION

Literature on carpometacarpal joint dislocations is scarce, and the subject represents just <1% of all hand injuries [6]. The first observations were described on the 19th century by Cooper & Roux [7]. Masquelet *et al.* [8] gathered 215 ases published in 1986, most of them being individual cases. The most numerous series are: 26 cases from Guimaraes *et al.* [9], 30 from Gerard *et al.* [10], and 31 from Gangloff *et al.* [11]. Among the rare dislocations of the four last metacarpals, those of the fourth and fifth metacarpals are more frequent than those of the second and third metacarpals due to the higher range of mobility of the former. The coexistence of a distal carpal row fracture is quite frequent (26%) [12].

The ligament and skeletal anatomy of the second through the fifth CMC joints has not been well described in the literature. Recognizing the inconsistent reports of the ligament anatomy, Nakamura *et al.* [13] performed an extensive and decisive study of 80 cadaver wrists to delineate and describe the ligament anatomy of this area. The diagnosis of this kind of injury is not always easy due to the large edema found in the hand and the difficulty to properly see the carpometacarpal joints on radiographies. Swelling around the wrist with shortening of the knuckle should alert the clinician towards the possibility of such an injury. On routine anteroposterior view, overlapping of joint surfaces, loss of parallelism, and asymmetry at the carpometacarpal joints should raise suspicion of the possibility of a subtle carpometacarpal injury. It is essential to perform radiological projection in pure oblique and lateral positions in order to be able to diagnose these kinds of injuries [14]. The treatment consists in the closed reduction when possible and fixation of Kirschner wires in cases of unstable injuries. When facing large open wounds or when it is impossible to reduce the dislocation in a closed way, it is necessary to resort to an open reduction. To the best of our knowledge, this is one of the few cases in which a volar dislocation of the three middle metacarpals linked with a Bennett’s fracture is described. The mechanism of injury in our case could have been a direct thrust over the knuckles that could have forced the metacarpals to rotate from the dorsal to the volar direction, causing dislocation of the middle three carpometacarpal joints and the Bennet’s fracture.

It is a kind of injure which, when treated acutely through reduction and fixation of Kirschner wires, tends to have a very low percentage of complications [15].

Among the main complications described in literature, cases of affected deep branch of the ulnar nerve [16], acute carpal tunnel syndrome [17] and avulsion of the extensor radial tendon of the carpus can be found.

In cases that are diagnosed late, the most frequent complication is a significant reduction of the mobility and strength of the fingers. Therefore, it is important to perform all the necessary radiological projections so as to diagnose these kinds of injuries as early as possible and treat them in the most accurate way. Intensive postoperative physiotherapy is vital for achieving a satisfactory outcome [18].

## Figures and Tables

**Fig. (3a):**
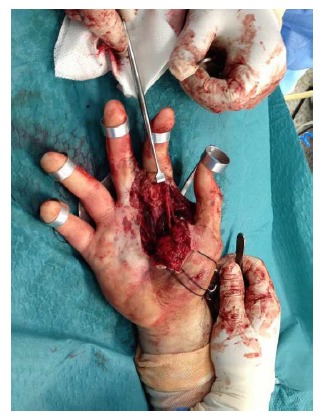
In this intra-operative image there can be seen the preservation of the superficial palmar arch of the hand which seems integral and uninjured.

**Fig. (3b):**
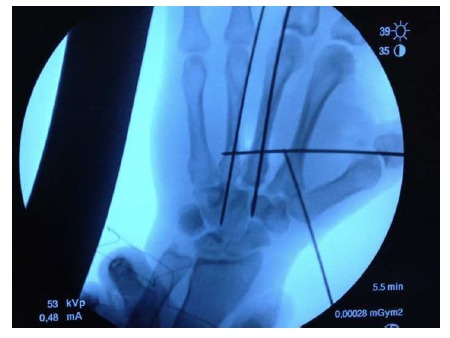
Radiological intra-operative image in which there can be seen the disposition of the Kirschner wires: one of them oblique from the base of the first to the second metacarpal, another transversally and two longitudinally following the axis of the central metacarpals.

**Fig. (3c):**
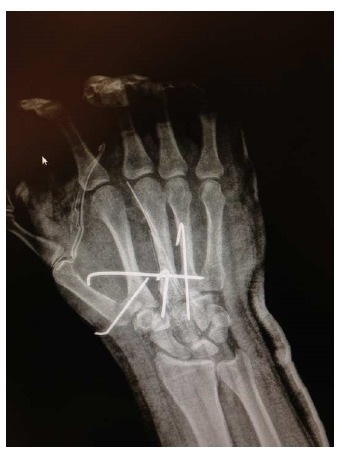
Post-operative radiographs showing the reduced second, third, and fourth carpometacarpal joints.
